# Small interfering RNA targeting NF-κB attenuates lipopolysaccharide-induced acute lung injury in rats

**DOI:** 10.1186/s12899-016-0027-y

**Published:** 2016-12-28

**Authors:** Ning Li, Yuanbin Song, Wei Zhao, Tingting Han, Shuhui Lin, Oscar Ramirez, Li Liang

**Affiliations:** 1 Department of Neonatology, Dongguan Children Hospital, Guangdong Medical University, Dongguan, China; 2 Pediatric Center of Zhujiang Hospital, Southern Medical University, Guangzhou, China; 3 Yale Cancer Center, Yale University School of Medicine, New Haven, CT USA; 4 Department of Pathology, Nanfang Hospital, Southern Medical University, Guangzhou, China

**Keywords:** Acute lung injury, Lipopolysaccharide, Nuclear factor-κB, RNA interference

## Abstract

**Background:**

To investigate the anti-inflammatory effects of specific small interfering RNA targeting NF-κB on lipopolysaccharide (LPS)-induced acute lung injury (ALI) in rats.

**Method:**

Acute lung injury was induced in Sprague-Dawley rats by intraperitoneal injection with LPS (5 mg/kg), followed by immediate intratracheal instillation of siRNA targeting NF-κB p65 (40 μg/ml). Animals in each group were sacrificed at 1 h or 8 h after the instillation. Pulmonary histological changes were evaluated by hematoxylin-eosin staining. The levels of NF-κB and TNF-α were measured by qRT-PCR. Expressions of NF-κB in lung cells and TNF-α in bronchoalveolar lavage fluid (BALF) were determined by western blot analysis and enzyme-linked immunosorbent assay (ELISA) respectively.

**Results:**

LPS administration reduced the rectal temperature and white blood cell counts at 1 h, increased lung wet/dry weight ratios, caused evident lung histopathological injury, and increased the detectable transcript and cytokine levels of TNF-α in lung tissue in BALF. siRNA targeting of NF-κB p65 effectively abrogated the expression of NF-κB p65 in lung cells and, aside from rectal temperatures, ameliorated all changes induced by LPS.

**Conclusions:**

NF-κB knockdown exerts anti-inflammatory effects on LPS-induced ALI especially in the initial phase, which may be due in part to reduced levels of the proinflammatory cytokine TNF-α. NF-κB siRNA’s rapidity and effectiveness to abrogate ALI development may provide an effective therapeutic method with future clinical applications.

## Background

Acute lung injury (ALI) and its severe manifestation, acute respiratory distress syndrome (ARDS), are well-defined clinical disorders characterized by severe hypoxemia, pulmonary edema and neutrophil infiltration. Among many clinical insults, sepsis represents one of the main cause of ALI. Unfortunately, the significant breakthrough in ALI diagnosis and therapy strikes a sharp contrast with its high morbidity and mortality [13]. This discrepancy can only be eliminated through the discovery of novel and effective pharmacological methods, which remain unsatisfactorily absent, especially in the initial phase of ALI.

Nuclear factor kappa B (NF-κB) is an evolutionarily conserved family of DNA binding proteins involved in transcriptional regulation of many gene products. Activation of the NF-κB pathway is a trigger that may initiate an inflammatory cascade and leads to the upregulation of many pro-inflammatory cytokines [15]. NF-κB regulates the expression of these cytokines by directly binding to the consensus sequences in their enhancer/promoter regions [5, 6, 8]. In addition, activation of NF-κB can be induced in response to lipopolysaccharide (LPS), tumor necrosis factor-α (TNF-α), interleukins, radiation and other stimulating agents. Importantly, the activation of NF-κB is observed in alveolar macrophages from patients with ARDS [18], indicating the implication of NF-κB pathway in the development and progression of ALI and ARDS. Therefore, determining whether pharmacologic inhibition of the NF-κB pathway inhibits the development and progression of ALI may provide a novel more effective therapeutic option for treatment of this disease.

Many attempts to target various key components of the classical activation pathway have been made in recent years. These include the inhibition of ubiquitination and proteosomal degradation of inhibitor of kappa B (IκB) and blocking of activated NF-κB’s binding to DNA but proved to be impractical in a clinical setting due largely to lack of specificity or necessity of pretreatment before insults. siRNA with high specificity, however, can target and decompose the complementary NF-κB mRNA [9], making it a perfect candidate for inhibiting the initially inflammatory cascade during ALI development via inhibition of NF-κB pathway activation.

In the present study, we undertook the challenge of determining whether targeted depletion of NF-κB could block the development and progression of LPS-induced ALI in rats without the necessity of pretreatment. Moreover, we wanted to determine how inactivation of the NF-κB pathway contributed to the suppression of the inflammation. Our results show that siRNA depletion NF-κB is directly responsible for decreased levels of TNF-α and reduces the pathology of ALI.

## Methods

### Animals use approval and reagents

Sprague-Dawley rats weighing 100–150 g were purchased from the Experimental Animal Center of the Southern Medical University (Guangzhou, China). All animals were allowed food and tap water ad libitum and exposed to a 12 h light/12 h dark cycle in accordance with the Principles of Laboratory Animal Care approved by Southern Medical University. LPS (O111:B4) was purchased from Sigma Chemical Company (St. Louis, MO., USA) and dissolved in 0.9% saline before use. SYBR Premix Taq™ kit was purchased from TaKaRa Biotechnology Co., LTD (Shiga, Japan). Tissue protein extraction reagent was purchased from TaKaRa Biotechnology Co., LTD (Shiga, Japan). Antibody specific for total NF-κB p65 subunit was purchased from Abcam (Cambridge, MA., US). Enzyme-linked immunosorbent assay (ELISA) kit of TNF-α was purchased from Thermo Scientific Pierce Protein Research Products (Rockford, IL., USA).

### RNA interference

Three siRNAs targeting NF-κB were synthesized by Shanghai GenePharma Co., LTD (Shanghai, China). All siRNAs (sense: 5′-GGA GUA CCC UGA AGC UAU AUU-3′; antisense: 5′- UAU AGC UUC AGG GUA CUC CUU -3′) were tested on lung tissue cells to choose the one with the highest gene silencing efficacy for further use. The scrambled siRNA (sense: 5′-UUC UCC GAA CGU GUC ACG UUU-3′; antisense: 5′-ACG UGA CAC GUU CGG AGA AUU-3′) was used as control. All siRNAs were dissolved by DEPC-treated water to a final concentration of 40 μg/ml.

### Construction of LPS-induced ALI models

According to individual weights, SD rats were intraperitoneally injected with 5 mg/kg LPS dissolved in 2 ml sterile saline with a 20 gauge-needle syringe to establish ALI models. Since LPS administration can cause neutrophil infiltration, interstitial edema, hemorrhage and proinflammatory cytokine production, which effectually simulates the natural development of ALI, it provides us with an optimal platform to test siRNA efficacy.

### Experimental protocol

All SD rats were randomly assigned to one of the following four groups (*n* = 12, each group). Saline + DEPC group received an intratracheal instillation of 1.5 ml DEPC-treated water immediately after an intraperitoneal injection of 2 ml saline. LPS + DEPC group received an intratracheal instillation of 1.5 ml DEPC-treated water immediately after LPS administration. LPS + scramble group received an intratracheal instillation of 1.5 ml scramble RNA (40 μg/ml) immediately after LPS administration. LPS + siRNA group received an intratracheal instillation of 1.5 ml siRNA (40 μg/ml) immediately after LPS administration. Six rats in each group were randomly sacrificed at 1 h and 8 h after the instillation respectively. Rectal temperatures were measured before both LPS injection and then sacrifice. Rectal temperature changes were calculated by taking the rectal temperature at time of sacrifice and subtracting the rectal temperature before LPS injection. Right lung was lavaged to collect bronchoalveolar lavage fluid (BALF). Then the lower lobe of right lung was rapidly excised and preserved in the liquid nitrogen for subsequent tissue RNA and protein extraction. The superior lobe of left lung was used for histopathological examination. The lower lobe of left lung was excised for analysis of lung wet/dry weight ratios. Blood samples were collected from iliac artery. The rats were anesthetized by chloral hydrate (3.5 ml/kg) throughout the surgical procedures.

### Bronchotracho alveolar lavage (BAL)

Animals were anesthetized by intraperitoneal injection of chloral hydrate (3.5 ml/kg) and fixed on a board in a supine position. After sterilization, a median sternotomy was performed to expose both of lungs and trachea. The hilum of the left lung was ligated, and then the right lung was lavaged twice with 5 ml ice-cold phosphate buffered saline (PBS). Each time, 4.5 ml of the injected PBS was recovered. The collected BALF was immediately centrifuged at 500 × g for 10 min at 4 °C, and the cell-free supernatant were stored at -80 °C until TNF-α concentration assay. The concentration of TNF-α in BALF was quantified by enzyme-linked immunosorbent assay (ELISA) according to the manufacture’s recommendations.

### Histopathological examination

The superior lobe of left lung of each rat was harvested when rats were sacrificed, fixed in 10% neutral buffered formalin for 24 h, and embedded in paraffin. Tissues were cut into a series of microsections (4 μm), and then stained with hematoxylin-eosin using standard protocols.

### Lung wet/dry weight ratio

As an indication of lung edema, the lung wet/dry weight ratio was calculated by dividing the wet weight by the dry weight. The lower lobe of the left lung was excised and weighed to obtain the wet weight. After drying in an oven at 80 °C for 24 h, the lobe was weighed again to obtain the dry weight.

### Western blot analysis

The lower lobe of the right lung was harvested and stored in −80 °C liquid nitrogen. After homogenization of tissue samples, total proteins were extracted and their concentrations were measured by BCA protein assay. Cell lysates (20 μg protein/lane) were separated on 10% SDS polyacrylamide gel electrophoresis and transferred to polyvinylidene difluoride membranes, which were subsequently blocked with 5% skim milk in a PBST solution (100 mM NaCl,50 mM Tris,0.1% Tween-20,PH 7.5)for 1 h at room temperature and probed overnight at 4 °C with appropriate primary antibodies. Horseradish peroxidase (HRP) conjugated anti-rabbit IgG and anti-rat IgG were used as secondary antibodies according to the primary antibodies. Immunoreactive bands were visualized by enhanced chemiluminescence.

### Total RNA extraction and qRT-PCR

The lower lobe of the right lung was harvested and stored in −80 °C liquid nitrogen. Total RNA was extracted from tissue samples using Trizol reagent. 2 μl of total RNA from each sample in a final reaction volume of 10ul was converted into single-stranded cDNA using SuperScript II Reverse Transcriptase (Invitrogen) following the manufacturer’s recommendations. The RT PCR reaction was carried out with 37 °C for 15 min and 85 °C for 50 s. qRT-PCR was performed using SYBR Premix Taq™ kit with the 95 °C activation step for 30 s; 95 °C denaturation step for 5 s, 62 °C annealing step for 30 s (NF-κB) or 58 °C annealing step for 30 s (TNF-α), 72 °C extension step for 34 s for 40 cycles; and a final extension step of 72 °C for 4 min. The following primers were used: NF-κB forward: 5′-GGG ACT ATG ACT TGA ATG CGG TCC -3′, reverse: 5′-CAG GTC CCG TGA AAT ACA CCT CAA-3′; TNF-α forward: 5′-CTT CTG TCT ACT GAA CTT CGG-3′, reverse: 5′-GTG CTT GAT CTG TTG TTT CC-3′; GAPDH forward: 5′-ACC ACA GTC CAT GCC ATC AC-3′, reverse: 5′-TCC ACC ACC CTG TTG CTG TA-3′. Results in triplicate were expressed as relative transcriptions of the NF-κB mRNA and TNF-α mRNA using the following formula: $\mathrm{Folds} = {2^{\hbox{-}}}^{\varDelta \varDelta \mathrm{C}\mathrm{t}};\kern0.5em \varDelta \varDelta \mathrm{C}\mathrm{t} = {\left(\mathrm{C}{\mathrm{t}}_{{}_{\left(\mathrm{target}\ \mathrm{genes}\right)}}-\mathrm{C}{\mathrm{t}}_{{}_{\kern0.5em \left(\mathrm{GAPDH}\right)}}\right)}_{\mathrm{experimental}\ \mathrm{group}}-{\left(\mathrm{C}{\mathrm{t}}_{{}_{\left(\mathrm{target}\ \mathrm{genes}\right)}}-\mathrm{C}{\mathrm{t}}_{\left(\mathrm{GAPDH}\right)}\right)}_{\mathrm{control}}$.

### Statistical analysis

Statistical analysis was conducted using SPSS 13.0 software package. All data were expressed as mean ± S.D. and compared using the student’s *t*-test for two-group analysis and one-way analysis of variance (ANOVA) followed by Student-Newman-Keuls method for multiple-group analysis. Differences in values were considered significant at *P* < 0.05.

## Results

### Effects of NF-κB siRNA on pulmonary histopathological changes of LPS-induced ALI rats

**Fig. 1:**
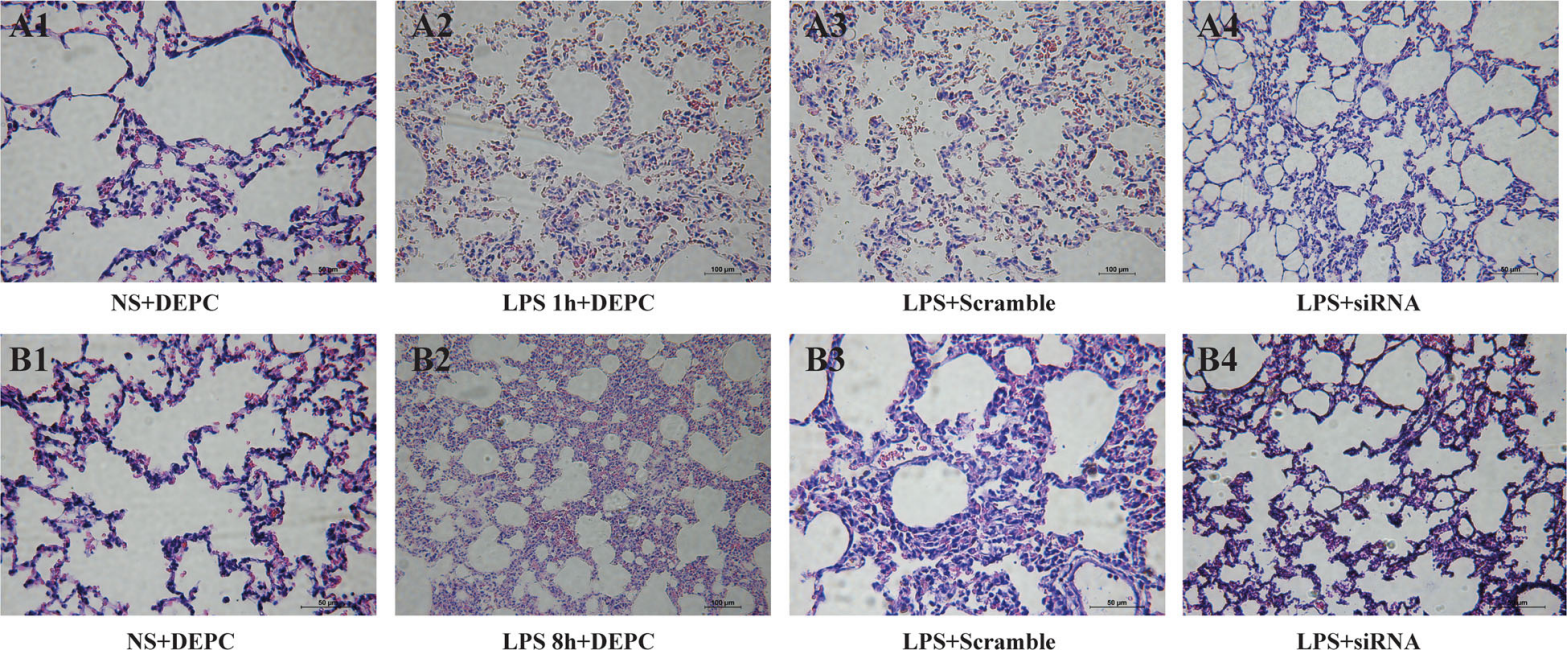
NF-κB siRNA preserves lung histology during LPS-induced ALI. Rats were treated with siRNA specific to NF-κB and lung micro-sections were stained with hematoxylin-eosin to assess histopathological damage at one or eight hours after LPS administration. Pulmonary histopathological changes of all groups 1 h after saline or LPS administration (**a**). Pulmonary histopathological changes of all groups 8 h after saline or LPS administration (**b**)

We established the lung injury rat model by LPS administration. Histopathological changes were characterized by widespread alveolar wall thickness caused by edema, severe hemorrhage in the alveolus, alveolus collapse and obvious neutrophil infiltration 1 h after LPS administration. Furthermore, histopathological changes worsened at 8 h after LPS administration. On the contrary, lung tissues from control group exhibited clear and normal alveolar structures (Fig. 1). We next evaluated the effect of NF-κB siRNA on pulmonary histopathological changes of LPS-induced ALI rats and found that immediately intratracheal instillation of NF-κB siRNA effectively attenuated histopathological damage at each time point (Fig. 1). These data showed that NF-κB siRNA exerts rapid and potent anti-inflammatory effects on lungs exposed to LPS.

### Effects of NF-κB siRNA on the expressions of NF-κB and NF-κB p65 in lung tissues of LPS-induced ALI rats

**Fig. 2:**
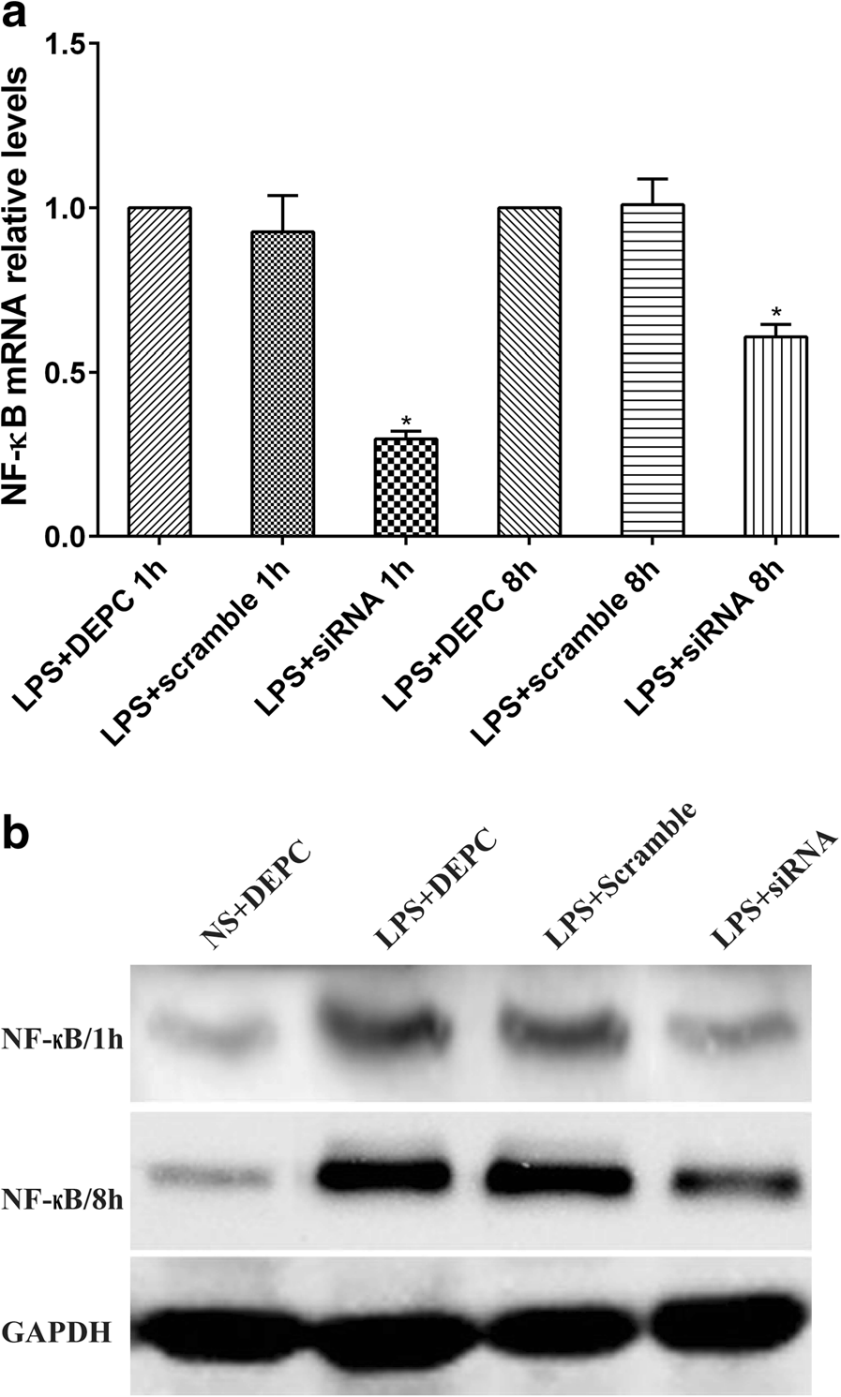
NF-κB siRNA administration decreased NF-kB transcript and protein levels in lung tissues of rats with ALI. **a** qRT-PCR was used to compare the levels of NF-κB mRNA transcription between LPS + scramble group and LPS + siRNA group. The amplification of GAPDH was used as internal control. Data were expressed as mean ± S.D. from three individual experiments. *P < 0.05 vs. LPS+. **b** Rats were treated with siRNA specific to NF-κB for one or eight hours, lung tissue was collected and processed by western blot analysis to determine the levels of NF-κB p65 expression after one or eight hours

We detected the levels of NF-κB mRNA and NF-κB p65 in lung tissues of LPS-induced ALI rats by q-RT-PCR and Western blot, respectively. At 1 h after LPS exposure, the level of NF-κB mRNA transcription in LPS + siRNA group was significantly reduced (0.297) compared with that in LPS + scramble group (0.927). Similarly, at 8 h after LPS exposure, NF-κB transcript levels in LPS + siRNA group was significantly reduced (0.607) (Fig. 2a). LPS administration caused a notable increase in NF-κB p65 protein levels compared with control group at each time point. However, in siRNA group, immediately following intratracheal instillation of NF-κB siRNA markedly suppressed NF-κB p65 expression. There were no significant differences of NF-κB p65 expression between LPS + DEPC group and LPS + scramble group at each time point (Fig. 2b). Thus, the intratracheal instillation of NF-κB siRNA led to the strong knockdown of NF-κB in lung tissues of LPS-induced ALI rats.

### Effects of NF-κB siRNA on the rectal temperature, white blood cell counts, and lung wet/dry weight ratio of LPS-induced ALI rats

**Fig. 3:**
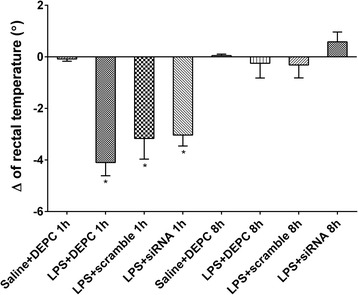
NF-κB siRNA changes rectal temperature after LPS administration. Rectal temperature was measured after ALI induction and changes tracked at zero, 1 or 8 h. Changes in rectal temperature were calculated at each time point and presented as the mean of differences in temperature. Data were expressed as mean ± S.D. (*n* = 12). *P < 0.05 vs. saline + DEPC

**Fig. 4:**
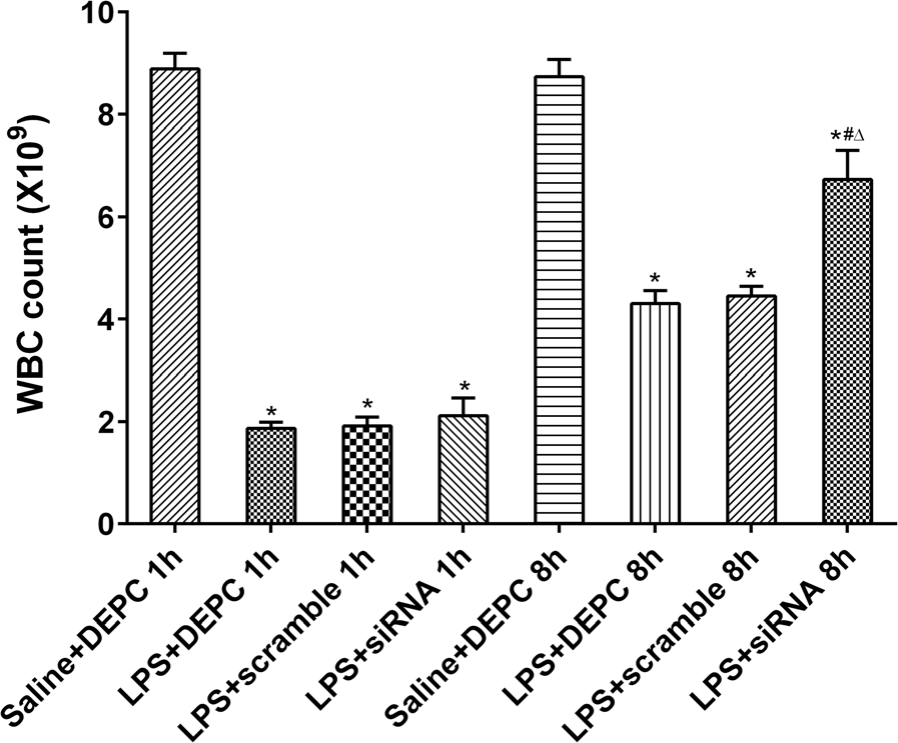
NF-κB siRNA alters white blood cell counts after LPS administration. Rats were treated with siRNA specific to NF-κB; blood was collected and processed to assess total WBC. Data were expressed as mean ± S.D. (*n* = 12). *P < 0.05 vs. saline + DEPC;#*P* < 0.05 vs. LPS + DEPC;△*P* < 0.05 vs. LPS + scramble

**Fig. 5:**
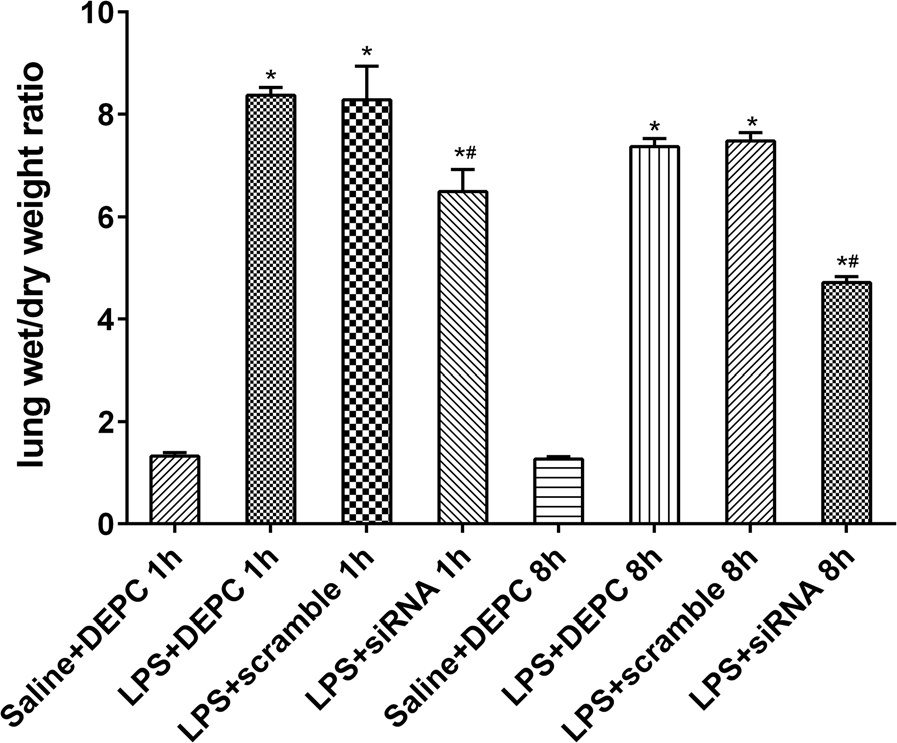
NF-κB depletion alters lung wet/dry weight ratios after LPS administration. Rats were treated with siRNA specific to NF-κB and lung tissue was collected and processed to assess the wet to dry ratio at one or eight hours. Data were expressed as mean ± S.D. (*n* = 12). *P < 0.05 vs. saline + DEPC;#*P* < 0.05 vs. LPS + DEPC;△*P* < 0.05 vs. LPS + scramble

We further examined whether NF-κB siRNA influenced rectal temperature, white blood cell counts, and lung wet/dry weight ratio in LPS-induced ALI rats. The rectal temperature of rats was significantly decreased 1 h after LPS administration compared with control group. In contrast, no significant differences among all groups at 8 h were observed. These results indicate that rectal temperature could fully recover within 8 h after the onset of LPS-induced ALI. Immediate intratracheal instillation of NF-κB siRNA had minimal effect on the changes induced by LPS (Fig. 3). LPS administration significantly reduced white blood cell counts at each time point. At 1 h after LPS administration, immediately intratracheal instillation of NF-κB siRNA did not efficiently increase white blood cell counts. In contrast, at 8 h after LPS administration, it significantly increased white blood cell counts. There were no significant differences in white blood cell counts between LPS + DEPC group and LPS+ scramble group at each time point (Fig. 4). Compared with control group, the lung wet/dry weight ratio was obviously enhanced after LPS administration at each time point. Rats with NF-κB siRNA instillation had a reduced lung wet/dry weight ratio. No significant differences in the lung wet/dry weight ratio were observed between LPS + DEPC group and LPS+ scramble group at each time point (Fig. 5). The above results showed that NF-κB siRNA obviously increased white blood cell counts at 8 h and reduced lung wet/dry weight ratio in LPS-induced ALI rats.

### Effects of NF-κB siRNA on the level of TNF-α mRNA expression in lung tissues and concentration of TNF-α in BALF

**Fig. 6:**
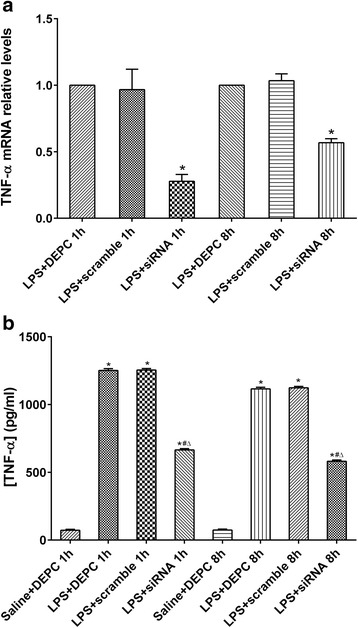
Specific targeting of NF-κB ablates TNF-α transcript and protein levels in lung tissue. Rats were treated with siRNA specific to NF-κB and TNF-α transcript levels were assessed at 1 and 8 h by qRT-PCR and compared between LPS + scramble group and LPS + siRNA group (**a**). The amplification of GAPDH was used as internal control. Data were expressed as mean ± S.D. from three individual experiments. #*P* < 0.05 vs. scramble group. Rats were treated with siRNA specific to NF-κB and TNF-α levels were assessed at one and eight hrs by standard ELISA (**b**). Data were expressed as mean ± S.D. (*n* = 12). *P < 0.05 vs. saline + DEPC;#*P* < 0.05 vs. LPS + DEPC;△*P* < 0.05 vs. LPS + scramble

Finally, we measured the level of TNF-α mRNA in lung tissue and TNF-α concentration in BALF by qRT-PCR and ELISA, respectively. At 1 h after LPS administration, TNF-α transcript levels in LPS + siRNA group were significantly reduced (0.278) compared with that in LPS + scramble group (0.967). Similarly, at 8 h after LPS administration, TNF-α transcript levels in LPS + siRNA group were significantly reduced (0.568) compared with that in LPS + scramble group (1.034) (Fig. 6a). LPS administration significantly increased the concentration of TNF-α compared with control group. Immediate intratracheal instillation of NF-κB siRNA partially reversed this increase. There were no significant differences in concentrations of TNF-α in BALF between LPS + DEPC group and LPS + scramble group at each time point (Fig. 6b). These data suggest that the anti-inflammatory effects observed after NF-κB’s knockdown may be directly associated with the decreased levels of TNF-α.

## Discussion

Recent years have seen a growth in our understanding of ALI/ARDS, thus leading to a substantial improvement of disease outcome upon pharmacological treatment. Sepsis serves as a leading predisposing factor of ALI and may be the direct result of the LPS on the outer membrane of various gram-negative bacteria. Therefore, LPS can be used to establish ALI animal models through intravenous injection, intraperitoneal injection and intratracheal instillation [6]. At the present time, clinical trials with the use of pharmacologic options abound and include but are not limited to the following [16]: 1, Anti-endotoxin and anti-cytokine therapy; 2, Anti-adhesion molecule therapy; 3, Alveolar surfactant replacement therapy; 4, treatment with glucocorticoids which attenuate fibrosis, among others. Despite numerous options, glucocorticoids are among the few that have attained clinical usage. However, their disappointing efficacy in the acute phase of ALI and increased risk of infection leaves much to be desired. In the present study, we undertook the challenge of identifying a novel strategy that was both effective in the early stages of ALI and devoid of side effects by using RNA interference.

Taking advantage of the siRNA technology that has been optimized and made highly efficient in the past decade; we substantiated that intratracheal instillation of NF-κB siRNA exerted rapid and potent anti-inflammatory effects on lungs exposed to LPS. The key of RNA interference (RNAi) lies in delivery and stable expression of siRNA and has been used to cure disorders of the eyes, lungs and central nervous systems [1, 2, 4, 20, 21]. Bitko et. al for the first time demonstrated that intranasal instillation of siRNA free of transfection reagents exerted a robustly anti-inflammatory effect on individual as well as joint infection by respiratory syncytial virus (RSV) and parainfluenza virus (PIV), and that when properly designed; direct siRNA administration turns out to be feasible in the treatment of lung diseases. Moreover, this method displays many advantages [1]. First, direct siRNA administration through the respiratory system is relatively simple, noninvasive, and compatible with an inhaler or mist based therapy. Second, the exclusion of a virus-based carrier in this method reduces the potential risk of carrier side effect. Therefore, we employed intratracheal instillation of siRNA to curb the NF-κB pathway activation. As shown in Fig. 2a and b, we were able to effectively target and significantly decrease NF-kB levels in our preclinical model. By using the highly specific siRNA approach, we were able to decrease the detectable levels of NF-kB after LPS administration in rat lungs at 1 and 8 h intervals. These results show that specific siRNA targeting of NF-kB can be accomplished and thus help establish and validate our preclinical model.

To further investigate the effectiveness on NF-kB knock down on lung histology, we treated rats with LPS, siRNA or control. Our data demonstrate that upon ALI induction followed by siRNA, the level of lung injury can be significantly altered (Fig. 1). In addition to decreasing lung injury, NF-kB knockdown also resulted in a remarkable decline in lung wet/dry weight ratio (Fig. 5). Multiple attempts have been made through the years to suppress ALI by blocking NF-κB pathway, including stabilization of IκBα [8, 9, 11, 12], inhibition of binding between activated NF-κB dimers and target genes via decoy oligonucleotides [3], and transcriptional inhibition of key components of the NF-κB pathway via antisense technology [17]. Recently, drugs such as butyrate [14] and pitavastatin [19] were found to be effective in preventing the initiation of ALI by interacting with NF-κB pathway. These attempts, however, have limited clinical merit primarily because they cannot specifically block the NF-κB pathway without impairing other cellular activities, leading to known and unknown side effects. Furthermore, the necessary pretreatment is also a bottleneck in clinical practice. In contrast, the high specificity of siRNA without the need of pretreatment in our study makes it a more ideal strategy compared with other methods. Taken together, our data represent a novel and highly effective way to specifically target NF-kB, reduce lung injury while minimizing detrimental and unwanted side effects.

Cells of the immune system are key producers of cytokines in response to a plethora of immunologic insults. NF-kB has been shown to be a key regulator of many immunologic responses [7]. To this end, we wanted to determine the effects of NF-kB targeting on total white blood cells counts. Indeed, knock down of NF-kB resulted in the amelioration of white blood cell counts 8 h after LPS administration (Fig. 4). Our results are in line with previously published reports and further provide evidence for the central role of NF-kB in ALI and immunity [9, 10].

Given its impact on immunologic function and cytokine production, we next wanted to assess whether NF-kB targeting would also alter cytokine production. As seen in Fig. 6a and b, NF-κB siRNA effectively suppressed levels of TNF-α mRNA transcription and concentrations of TNF-α in BALF, providing evidence to support the possibility that the mechanism of NF-κB siRNA’s anti-inflammatory effects is likely associated with the reduction of proinflammatory cytokines. TNF-α, however, can be regulated by other pathways, and should e further investigated but are beyond the scope of this manuscript [9].

## Conclusion

In conclusion, NF-κB specific knockdown exerts its anti-inflammatory effects on LPS-induced ALI likely because of a reduction of the proinflammatory cytokine, TNF-a. Therefore, we offer the following; NF-κB siRNA treatment represents an effective and novel strategy for modulating inflammatory response for ALI in the early stages with high clinical relevancy. One important point is that we acknowledge that a limitation of the study is the direct translational implications of our findings as NF-κB siRNA treatment in humans would represent a significant challenge given that clinical symptoms appear much later then the time points investigated here.

## Abbreviations

ALI: Acute lung injury
ANOVA: One-way analysis of variance
ARDS: Acute respiratory distress syndrome
BALF: Bronchoalveolar lavage fluid
ELISA: Enzyme-linked immunosorbent assay
HRP: Horseradish peroxidase
IκB: Inhibitor of kappa B
LPS: Lipopolysaccharide
NF-κB: Nuclear factor kappa B
PBS: Phosphate buffered saline
PIV: Parainfluenza virus
RSV: Respiratory syncytial virus
siRNA: Small interfering RNA
TNF-α: Tumor necrosis factor-α
